# Health diplomacy in Africa-opportunities post-COVID-19

**DOI:** 10.11604/pamj.2022.43.91.37003

**Published:** 2022-10-19

**Authors:** Hiba Boujnah

**Affiliations:** 1 Africa Centres for Disease Control and Prevention (Africa CDC), Addis Ababa, Ethiopia

**Keywords:** Health diplomacy, health policy, trade policy, regionalism, Africa

## Abstract

For the last seventy years, Africa has suffered a disease burden that is steadily growing in scale and complexity. Despite that, health development in the continent has continued to rely on donors´ packages since decolonization. The last decade, however, has marked some health-related achievements on the continent such as the development of the Africa Health Strategy 2016-2030, the establishment of Africa Centres for Disease Control and Prevention (Africa CDC), the launch of The African Continental Free Trade Area (AfCFTA) and most recently the African Medicines Agency (AMA). These developments and the response to the COVID-19 pandemic have highlighted the importance and the opportunities of practicing Global Health Diplomacy on the continent. Home to 27% of the world´s countries, Africa has a tremendous global voting power which makes global health diplomacy an unequivocally effective soft power tool to achieve “The Africa we want”. In this paper, we will expand on the importance of Global Health Diplomacy (GHD) practice in Africa as a soft power tool, illustrate the COVID-19 response in the continent championed by the Africa Centres for Disease Control and Prevention (Africa CDC) as a case study, and offer some recommendations to sustain and strengthen GHD´s role in the continent.

## Essay

### Introduction

As global health threats continue growing, health has shifted from low politics to high politics. It is increasingly recognized that “Health is everyone´s business”. Global Health Diplomacy (GHD) has then become an undeniable tool for 21^st^-century public diplomacy, especially against the backdrop of the COVID-19 pandemic and for countries that suffer the greatest disease burden. In this paper, we will focus on the importance of GHD practice in Africa as a soft power tool illustrating the COVID-19 response in Africa championed by the Africa Centres for Disease Control and Prevention (Africa CDC) as a case study, and we will offer some recommendations to sustain and strengthen GHD´s role in the continent.

### Global health diplomacy in Africa

In just three decades 1914-1945, Africa faced four major cataclysms between First World War, the Spanish Flu Epidemic in 1918, the 1929 economic crisis, and the Second World War. Despite the tremendous impact these crises had on health outcomes, the colonial powers did not favor a continental approach to health cooperation, and the efforts of the League of Nations Health Organization (LNHO) tended at best to build bilateral interactions which did not allow for continental health diplomacy [1]. This model pertained after 1945, despite the rise of multilateralism through United Nations (UN) institutions. Two different World Health Organization (WHO) regional offices were created to cover Africa: EMRO (1949) and AFRO (1951). Despite the continent high disease burden, AFRO´s planning capacity was “extremely limited” until the region's decolonization [1]. Given Africa's rising public health institutionalization movement, and in alignment with the *“new public health order*” adopted by the African Union (AU) and the Africa CDC, it is necessary to better understand the health policy space, its key players, and governance mechanisms of Global Health Diplomacy (GHD) [2]. Global Health Diplomacy (GHD) is a powerful negotiating tool that shapes global health policy, pulling expertise from public health, law, international affairs, management, and economics [3]. Worldwide, governments and decision-makers face complex global problems requiring borderless and innovative solutions. Health challenges are no exception and their solutions require the involvement of multiple stakeholders at different levels and, therefore, cannot be addressed without exercising GHD. Global Health Diplomacy (GHD) has excellent leverage on five global domains, namely (1) Global health security, (2) Strengthening health systems, (3) International cooperation and global solidarity, (4) Global economy and trade and development, and (5) Addressing inequities to achieve the global health targets [3,4]. Africa is the continent with the most countries in the world (27% of the world´s countries) [5]. Leveraging GHD as a soft power tool for negotiation offers Africa a great voting power (one person, one vote) and a global negotiation power, especially given the rise of Global Health Security in peace and security as well as in economic welfare.

### Why is addressing health diplomatically essential and timely in Africa?

**1) The disease burden in Africa**

The African continent, home to 15% of the world´s population, suffers more than 25% of the global disease burden [5]. For the last seventy years, Africa has suffered a continuous burden of illness, exacerbated by the COVID-19 pandemic [2] resulting in a syndemic of infectious and non-communicable diseases (NCDs), and nutritional disorders [6]. Recognizing that health threats will multiply as our habits and environment continue evolving, disease prevention remains essential and health system strengthening becomes paramount. Diseases thrive in times of political divisions and lack of cooperation [4]. Therefore, health diplomacy at large is essential for global action.

**2) Health as a soft power tool**

Today, Africa is the only continent in the world where official aid inflow outweighs private capital inflow by far [7]. Africa's health sector has long relied on development-related funds which hinders the health development [8]. Both the traditional North-South and the emerging South-South health cooperations have been a gateway to achieving non-health-related foreign policy objectives. However, South-South health cooperation allows a more symmetrical dynamic and better policy ownership for recipient countries [9]. On the other hand, continuing to consider health as a desired outcome of development is flawed since data showed health investments were correlated to rapid economic growth [10]. The spread of diseases increasingly threatens Africa's development [7], national security, trade, and economic agendas which are the building blocks of the AU Agenda 2063. These findings reverse the conventional wisdom: health is no longer simply a byproduct of growth but one of its engines. Traditionally, donors´ health packages allowed donors to serve their political and economic interests (bilateral and multilateral organizations, the private sector). The fact of relying on external funds to address regional health challenges in the absence of solid governance mechanisms exposed the continent to achieving agendas that might not be aligned with its own needs and vision. Donors´ health packages allowed funders to leverage soft power and gain relevance in negotiations on security, trade agreements, and development policies [11]. While African countries have benefited from donor packages, donor countries built on this to consolidate their influence and promote their interests. Conversely, health diplomacy has been relatively underused to advance African countries' interests. However, during the pandemic, African governments demonstrated great solidarity through the implementation of drastic policies and regionally orchestrated action to mitigate the human and socio-economic toll of COVID-19, especially on the informal sector, which employs over 85% of Africans. Days after the first COVID-19 confirmed case in the continent, on February 22^nd^, 2020, the AU's first public health specialized agency, Africa CDC, mobilized all the African health ministers for a consultation to respond to the pandemic.

Africa CDC was established recognizing the importance of the nexus between global health and broader goals in diplomatic efforts toward realizing Agenda 2063. Its mandate is to streamline AU Member States' public health efforts and strengthen their public health institutions' capacity in detecting, preventing, controlling, and responding to disease threats swiftly and effectively. In addition to Africa CDC, the last decade has marked powerful signs of continental solidarity such as the development of the Africa Health Strategy 2016-2030 and the incoming African Medicines Agency (AMA). However, the potential for health diplomacy in Africa remains largely untapped. Public health is still not considered a top priority in key agenda-setting documents such as the AU Agenda 2063. Before the inception of the Africa CDC, the lack of a unified African voice on health matters created a void that often leaves countries vulnerable to different influences. This could be illustrated with two events [11]: in adopting the political declaration of the high-level political declaration on universal health coverage in 2019, Africa missed a chance to display the collective negotiating power of its 55 states. Since countries signed separately, some African countries signed an alternative statement on universal health coverage initiated by the United States of America, which excluded sexual and reproductive health rights-a critical health challenge in Africa; at the 2019 World Health Assembly, 20 countries (including five African countries) sponsored a resolution to improve the transparency of markets for medicines, vaccines, and other health products. The resolution aims to provide governments with useful information allowing fair negotiations. The resolution was amended to lack some critical elements of transparency due to objections raised by Germany, Japan, the United Kingdom, and the United States, who opposed publishing production data and other relevant costs and subsidies from governments and other groups. There were no objections to the final text of World Health Assembly resolution WHA72.8. raised by African countries despite the continent´s lack of availability and access to vaccines, therapeutics, diagnostics, and other health commodities. It remains clear that the long history of colonialism and imperialism has shaped the perceptions and practices of GHD in Africa. As we continue moving forward, GHD should be increasingly considered and leveraged to bring more equality to cooperation and change the narrative of traditional foreign policy and commercial objectives on the continent [9].

**3) The African Continental Free Trade Area (AfCFTA)**

The AfCFTA aims to transform the African economy, which long lagged with the lowest continental internal trade in the world (17% in 2017, as opposed to Europe with 69% and Asia with 59% in the same year). This agreement entered into force on May 30^th^, 2019, just a few months before the pandemic. While the interconnectedness between trade and health goes back to the earliest human settlements, globalization has made that link stronger than ever before. Trade offers opportunities for better health but also threatens health outcomes when tensions arise between protecting health on one hand and generating wealth on the other. In a globalized world where trading powers and market capacities are not the same, special attention should be given to trading agreements and regulations of life-saving commodities as a matter of fairness and equity-a matter of global health that extends to peace and security. The absence of coherent trade agreements hinders reaching global health goals. It is then fair to say that trade is a health policy [12]. Therefore, if it does not account for any undesired implications on health, the AfCFTA will fail to improve Africans' life [13]. The AfCFTA´s opportunity to yield enormous economic and social growth for Africa could also cause harm in these exact domains according to its binding rules [14]. Numerous studies found that more open and free trade can be associated with: an increase in the availability and affordability of healthy foods, unhealthy foods, and other harmful products such as tobacco or alcohol results in increased trends in non-communicable diseases [14]; an improvement of health services quality, an increase in privatization of such services, including medical tourism, and a strain on access for low-income populations [14]. In the case of Africa, as demand for cancer treatment grows, visa-free travel will enable people in the 15 African countries without radiotherapy services to seek care elsewhere [15]; an increase in the migration of medical professionals from public to private institutions and from poorer to richer countries seeking better wages and working conditions. This results in weaker and understaffed public-health systems, especially in poorer countries [15]; a rise in the price of essential medicines with the enforcement of intellectual property rights [14]. The World Trade Organization (WTO) recognizes the substantial impact of trade policies on health. Trade policies such as tariffs, patent protection, and free trade have both direct and indirect effects on health. The following summarizes the findings of Fox and Meier (2007) [16] on four main trade agreements with direct effects and indirect effects that trade policy could have on health.

**A) Direct effects of trade policies**

1) Trade-Related Intellectual Property Rights (TRIPS). Forbids breaking pharmaceutical patents except via article XX(b) when “necessary to protect human, animal or plant life or health” TRIPS restricts access to generic medicines, resulting in higher drug prices, unaffordable for uninsured individuals and the developing world. 2) Agreement on the Application of Sanitary and Phytosanitary Measures (SPS). While recognizing the right of countries to take measures to protect health and life, SPS limits the use of these measures as trade barriers. 3) Regulatory standards governing human, animal, and plant health shall be based on recognized international bodies. Further restrictive regulation must be based on scientific risk assessment (for example, the EU ban on hormone-treated beef was deemed unsupported by science since it did not address defined risks). 4) Technical Barriers to Trade Agreement (TBT). Creates universal standards to protect human life and health, provided these standards are not surreptitious protective shields. Encourages the use of internationally agreed standards in product regulation. Regulations must be the least trade-restrictive necessary. (Technical barriers to trade has implications for water supply, food production, and labeling of foods and drugs). 5) General Agreements on Trade in Services (GATS). Establishes rules for service trade, including the movement of consumers and providers across borders to receive and supply health care with a view towards progressive liberalization. (may lead to privatization of health care).

**B) Indirect effects of trade policies**

Unjust trade laws hinder the economic development of developing countries and perpetuate the advantage of rich countries via: 1) non-tariff barriers to trade against exports from developing countries 2) the volatility of commodity prices. Trade liberalization has deprived countries of tariff revenue (a much-needed revenue stream in the developing world). Given the importance of health policy in trade policy, health diplomacy needs to play a central role in shaping the trading landscape in Africa, especially at such a pivotal moment in Africa´s history. Regional health diplomacy can achieve what single countries cannot. An illustrative example is a proposal for a temporary waiver on the Trade-Related Aspects of Intellectual Property Rights (TRIPS) in the World Trade Organization (WTO) drafted by India and South Africa to address COVID-19 therapeutics access for developing nations (Adetiba, 2021). If the TRIPS waiver request is accepted, access to vital COVID-19 drugs, technologies, and diagnostics can significantly improve. Therefore, strategic GHD is needed [3]. Africa could and should play a leading role in this transformative process. To conclude, as Africa implements the AfCTA, it is vital to closely engage with the AU health specialized agencies (Africa CDC and AMA) to ensure that trade-related health concerns are addressed [2].

**4) African Medicine Agency (AMA)**

African medical agency is the evolution of the African Medicines Regulatory Harmonization (AMRH) Initiative. Officially launched on November 5^th^, 2021, AMA is the second Africa Union (AU) specialized health agency following Africa CDC. African medical agency is mandated to ensure the strengthening of the continent's capacity in medicines regulation and the achievement of regulatory harmonization to improve access to safe, effective, and quality-assured medicines. The success of AMA requires strategic health diplomacy on both the regional and global levels, as explained by Margareth Ndomondo-Sigonda, Head of Health Unit at the African Union Development Agency - New Partnership for Africa´s Development (AUDA-NEPAD) [17]. For AMA to deliver its tremendous mandate, Member states need to understand the value of AMA, establish national regulatory agencies, and strengthen existing ones. AMA´s continental work needs to be implemented by closely engaging the Regional Economic Communities (RECs), and the Africa CDC´s regional structure (regional coordinating centres) should be used as a model [16]. This is another opportunity to make African regionalism a success. Furthermore, as AMA is modeled after the European Medicines Agency (EMA), GHD is necessary to guide the negotiations between the AU and the European Union on availing infrastructural, technological, administrative, and regulatory support to establish AMA [5].

### Health diplomacy in Africa case study: Africa CDC responds to COVID-19

Despite missed opportunities, the COVID-19 crisis has offered an exceptional opportunity to strengthen health diplomacy´s role in Africa. The AU Member States, with the support of the Africa CDC and the Regional Economic Communities (RECs), such as the Southern African Development Community (SADC), the Economic Community of West African States (ECOWAS), and the East African Community (EAC), have coordinated their pandemic responses, policies, and guidelines. Africa CDC has pioneered thirteen COVID-19 continental initiatives to date [18].

**1) The African Union COVID-19 response fund:** this fund aims to raise resources to strengthen the continental response to COVID-19. The fund has played a crucial role in financing the pandemic's response to vaccine purchasing and capacity building.

**2) The Africa joint continental strategy for COVID-19:** this strategy aims to prevent severe illness and death from COVID-19 infection and minimize the social disruption and economic consequences of COVID-19 outbreaks in a continentally coordinated manner.

**3) The Africa Task Force for Novel Coronavirus (AFTCOR):** the task force supports pan-African cooperation and African leadership in sharing information and best practices, building technical capacity, making high-quality policy decisions, and coordinating border detection and control.

**4) The Partnership to Accelerate COVID-19 Testing (PACT):** launched to promptly enhance the testing, tracing, and treating of COVID-19 cases to minimize the pandemic impact on the African continent.

**5) The Africa Medical Supplies Platform (AMSP):** the AMSP is a platform that unlocks quick access to an African & global base of vetted manufacturers and strategic procurement partners and enables the AU member states to purchase certified medical equipment and vaccines with increased cost-effectiveness & transparency.

**6) The Consortium for COVID-19 Clinical Vaccine Trials (CONCVACT):** aims to accelerate progress on COVID-19 vaccine trials in Africa through partnerships with relevant institutions and networks.

**7) Saving Lives and Livelihoods (SLL):** SLL is a partnership between the Africa CDC and mastercard foundation focusing on: access to COVID-19 vaccines for millions of Africans; strengthen member states' vaccine rollout capacity; strengthen African institutions to drive broader impact; ensure national, regional, and global health security.

**8) The African Vaccine Acquisition Task Team (AVATT):** provides a pooled procurement mechanism for vaccine purchasing and equitable distribution in the member states according to their populations´ size.

**9) The Partnerships for African Vaccine Manufacturing (PAVM):** an initiative to build vaccine manufacturing capacity on the continent, with 60% of vaccines needed to be manufactured locally.

**10) The Africa Pathogen Genomic Initiative (Africa PGI):** it is a four-year public-private partnership with US $100 million, aiming to expand access to next-generation genomic sequencing tools and expertise to strengthen public health surveillance and laboratory networks across Africa.

**11) The African Union trusted travel portal:** a simplified and secure system of vaccine records and PCR test results display at entry points.

**12) Saving lives, economies, and livelihoods:** a campaign that supports the development of a harmonized strategy to protect borders, travelers, economies, livelihoods, and schools in Africa mitigating COVID-19 risks using digital solutions.

**13) Bingwa initiative:** a public-private-youth initiative that seeks to establish a network of COVID-19 vaccination youth champions across the continent to advocate for COVID-19 vaccination in Africa.

### Proposed recommendations for global health diplomacy in Africa

In developing and strengthening strategic health diplomacy in Africa, African policymakers may like to consider the following actions [11]: institutionalize the integration of health into foreign policy. Governments should galvanize their efforts and adopt common positions, leading to resolutions suitable to African realities. A broader continental collaboration on health matters should materialize in joint guidelines, pooled resources, a readily deployable continental network of the public health workforce, and knowledge management platforms where best practices are exchanged. Leverage the existing regional blocs´ structure in Africa (RECs) in managing complex partnerships towards protecting Africa´s interests. Place Africa´s health agenda at the forefront and unify voices to exercise greater purchasing power and access innovative life-saving commodities. Increase the linkage between diplomats and health experts to ensure that health impacts are dully considered in negotiations through more inclusiveness and transparency in health-related decision-making and decision-taking processes.

Proactively create a dialogue at the intersection of health and foreign policy. It is now well recognized that national security depends on health security. Further, the pandemic has highlighted the link between domestic and foreign policy, whereby diseases do not know borders. Policy-makers must be well-informed of international health agreements and anticipate mitigating risks or threats. Negotiation processes for foreign policy and development agreements (including trade and commerce) should consider health agendas. Develop a cohort of African health diplomats and create a cross-learning between traditional diplomats and public health professionals through mutual placements at the diplomacy hubs (in Addis Ababa, Geneva, and New York) within ministries of foreign affairs and relevant AU entities (AU Department of Health Humanitarian and Social Affairs, Africa CDC, AMA, AfCFTA). African leaders should consider cross-disciplinarity in strengthening GHD and the “Health in All Policies” approach on the continent. The Kofi Annan Global Health Leadership Program is a good example, whereby emerging public health leaders are trained to work in political spheres and advocate to invest in health matters despite the competing emergencies on the continent [2].

Establish partnerships based on trust and mutual respect in accordance with the *“New Public Health Order”* to ensure partners´ efforts are aligned with the continental aspirations under the AU Agenda 2063 [2]. Such coordination is critical to minimize the duplications and inefficiencies that have long undermined the African-driven agendas. Harmonizing stakeholders´ efforts around Africa´s priorities must be at the core of the African Health Diplomacy. This committed continental engagement is necessary to establish an “Africa Disease Threat Fund”, inspired by The Global Fund´s model, to continue carrying forward Africa´s New Public Health order [2].

## Conclusion

Africa is the second most populated continent in the world. A healthy Africa is essential for the world´s growth, peace, and prosperity. As African leaders continue building the capacity of the continent, it is crucial to center their strategies around health and to collaborate with the international community as one continent. Global Health Diplomacy offers an unprecedented opportunity for a more equitable, healthier, and safer world for Africa and all.
